# Supplementary material for: Mutations in PCYT2 disrupt etherlipid biosynthesis and cause a complex hereditary spastic paraplegia
Source: Brain. 2019 Oct 22;142(11):3382–97. doi: 10.1093/brain/awz291 (PMC6821184; doi:10.1093/brain/awz291)
Supplement: awz291_Supplementary_Data [file awz291_supplementary_data.zip › awz291-Suppl_data/Supplementary_Data4.zip!/Supplementary information 4 - plasma lipidomics.zip!/Plasma lipidomics/Control vs Patient1/Statistical plots and data/PE[O] species/Boxplots.pdf]

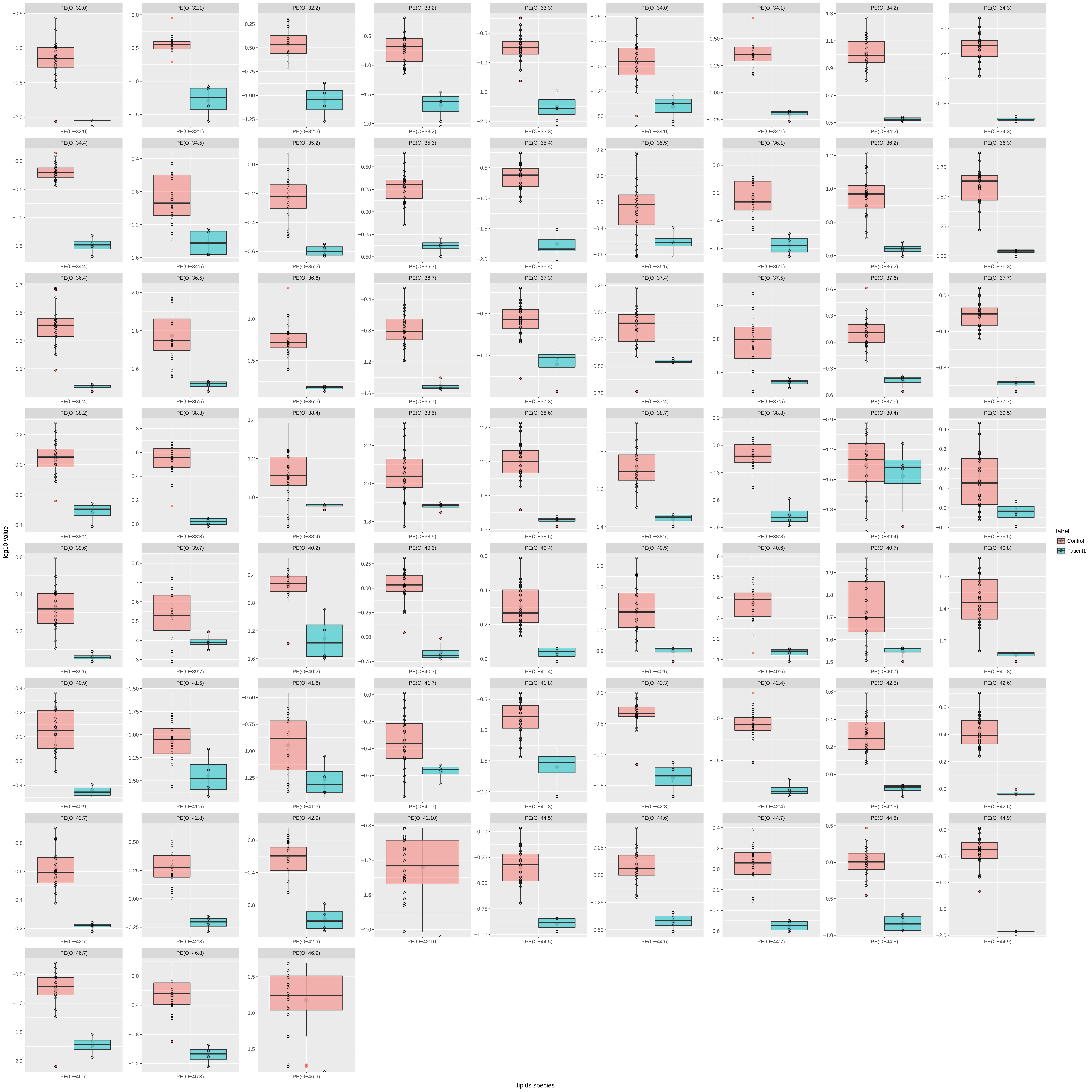
PE(O-32:0)
PE(O-32:1)
PE(O-32:2)
PE(O-33:2)
PE(O-33:3)
PE(O-34:0)
PE(O-34:1)
PE(O-34:2)
PE(O-34:3)
PE(O-34:4)
PE(O-34:5)
PE(O-35:2)
PE(O-35:3)
PE(O-35:4)
PE(O-35:5)
PE(O-36:1)
PE(O-36:2)
PE(O-36:3)
PE(O-36:4)
PE(O-36:5)
PE(O-36:6)
PE(O-36:7)
PE(O-37:3)
PE(O-37:4)
PE(O-37:5)
PE(O-37:6)
PE(O-37:7)
PE(O-38:2)
PE(O-38:3)
PE(O-38:4)
PE(O-38:5)
PE(O-38:6)
PE(O-38:7)
PE(O-38:8)
PE(O-39:4)
PE(O-39:5)
PE(O-39:6)
PE(O-39:7)
PE(O-40:2)
PE(O-40:3)
PE(O-40:4)
PE(O-40:5)
PE(O-40:6)
PE(O-40:7)
PE(O-40:8)
PE(O-40:9)
PE(O-41:5)
PE(O-41:6)
PE(O-41:7)
PE(O-41:8)
PE(O-42:3)
PE(O-42:4)
PE(O-42:5)
PE(O-42:6)
PE(O-42:7)
PE(O-42:8)
PE(O-42:9)
PE(O-42:10)
PE(O-44:5)
PE(O-44:6)
PE(O-44:7)
PE(O-44:8)
PE(O-44:9)
PE(O-46:7)
PE(O-46:8)
PE(O-46:9)
log10 value
lipids species
label
Control
Patient1
